# The Comparative Performance of Phytochemicals, Green Synthesised Silver Nanoparticles, and Green Synthesised Copper Nanoparticles-Loaded Textiles to Avoid Nosocomial Infections

**DOI:** 10.3390/nano12203629

**Published:** 2022-10-16

**Authors:** Muhammad Farrukh Tahir, Muhammad Zaman Khan, Safira Attacha, Noreen Asim, Muhammad Tayyab, Azam Ali, Jiri Militky, Blanka Tomková

**Affiliations:** 1 Department of Biochemistry, University of Jhang, Jhang 35200, Pakistan; 2 Department of Material Engineering, Technical University of Liberec, 46015 Liberec, Czech Republic; 3 Institute of Biotechnology and Genetic Engineering, The University of Agriculture Peshawar, Peshawar 2500, Pakistan

**Keywords:** phytochemicals, silver nanoparticles, copper nanoparticles, cotton fibre bandages

## Abstract

In the current study, a sustainable approach was adopted for the green synthesis of silver nanoparticles, green synthesis of copper nanoparticles, and the investigation of the phytochemical and biological screening of bark, leaves, and fruits of Ehretia acuminata (belongs to the family Boraginaceae). Subsequently, the prepared nanoparticles and extracted phytochemicals were loaded on cotton fibres. Surface morphology, size, and the presence of antimicrobial agents (phytochemicals and particles) were analysed by scanning electron microscopy, dynamic light scattering, and energy-dispersive X-ray spectroscopy. The functional groups and the presence of particles (copper and silver) were found by FTIR and XRD analyses. The coated cotton fibres were further investigated for antibacterial (qualitative and quantitative), antiviral, and antifungal analysis. The study revealed that the herb-encapsulated nanoparticles can be used in numerous applications in the field of medical textiles. Furthermore, the utility of hygienic and pathogenic developed cotton bandages was analysed for the comfort properties regarding air permeability and water vapour permeability. Finally, the durability of the coating was confirmed by measuring the antibacterial properties after severe washing.

## 1. Introduction

Healthcare-associated infections (HAIs), also called hospital-acquired infections (HAIs) or nosocomial infections (NI), develop in patients during hospitalisation and are a continuing concern within hospitals. The risk of these infections is on the rise despite the efforts to control them. These infections may cause disability or preventable death in humans [1,2]. Contaminated textiles (containing bacteria and viruses) might be a major source of cross-infection. The most common textile contributions in hospitals (wards, operation theatres, rooms, ICUs, and surgical areas) include bed sheets, pillow covers, outlets, surgical drapes, covering, panels, curtains, patient gowns, doctors’ gowns and socks, etc. [3]. The textiles are composed of natural fibres (which contain voids in structure, porosity, moisture, and natural contents) and are an excellent place for the growth, replication, and survival of pathogens [4]. 

Typically, pathogenic microorganisms such as *Escherichia coli*, *Staphylococcus Aureus*, vancomycin-resistant *Enterococci,* and *Clostridium* have been found on hospital textile surfaces [5,6]. The presence of these microbes on hospital textiles contributes to the spread of infections in the hospitalised patients and the staff. Additionally, bacterial strains (after *Ethicillin,* now Methicillin-resistant *Staphylococcus Aureus*) and virulent strains (the new SARS-CoV-2 strain) have developed resistance to last-resort drugs [7]. The rate of transmission of new virulent strains is very high and becomes catastrophic when an infected individual continuously spreads respiratory droplets on surfaces and has close contact with others [8,9,10]. The easiest way to evade harmful pathogens is to inhibit or kill them prior to their transmission inside the human body. For this reason, it is necessary to select suitable antiviral/antibacterial materials for use in daily life [9].

Researchers have been focused on the use of various kinds of antimicrobial finishings on textiles based on inorganic metal oxides [8]. The nano/microparticles of MgO, ZnO, Cu, Ag, TiO_2_, Cu_2_O, and CuO, etc., are of particular interest amongst them [7,10]. Copper-based antibacterial finishes are dependent specifically on their sizes and shapes to make sure there is a uniform particle size distribution on the textile substrate. Recently, both cuprous oxide and copper nanoparticles have gained a lot of attention in different potent applications, such as heat transfer systems, conductive inks or cooling fluids, catalysis, antiviral, antifungal, and antibacterial agents [11]. Azam et al. tried to coat the cotton fabrics for achieving antimicrobial activity through copper nanoparticles. The copper nanoparticles in nano-size are more susceptible to oxidation and conversion to the toxic carbonates and oxides [12,13].

The antimicrobial polymers based on nano-silver have gained considerable attention in recent years [14,15]. Silver is an inorganic, non-toxic metal which is a strong agent having the ability to kill 650 organisms (disease-causing) inside the body [15]. It is worth mentioning that silver exhibits wide antibacterial efficacy with low toxic effects for mammalians. Synthesis of silver nanoparticles through synthetic reducing agents is normally associated with biological hazards or environmental toxicity. In a similar way, another study was conducted to show the antimicrobial behaviour of silver nanoparticles. Due to the different environmental conditions, the particles were tarnished, and also caused toxicity and hazardous health problems [16]. 

Furthermore, researchers have been treating the infections of wounds using various antibiotics, such as meningitis, pneumonia, and tuberculosis in particular. However, microorganisms exhibit resistance towards present and synthetic antibiotics, so there is high demand to develop novel antibiotics that are cost-efficient and have easy availability in the market. Hence, there is a need to focus on more hygienic, biocompatible, and cost-effective green antibacterial and antibiotic agents. The plant-based extracts of *E. acuminata* leaves have been studied against various strains of bacteria to analyse their zone of inhibition and MIC. The methanolic extract showed excellent antibacterial activity against *Azospirillum lipoferum*, *Pseudomonas aeruginosa*, *Enterococcus sp*., *Stenotrophomonas maltophilia,* and *E. coli*, respectively, having MIC in the range of 0.8–5.1 mg/mL and a zone of inhibition in the 10.2–29 mm range [17]. Multiple studies were conducted on the green synthesis of different nanoparticles such as silver, zinc, etc., from different plant materials, including *Cinnamon zeylanicum* and *Cacumen Platycladi*. The bio-synthesised nanoparticles exhibit significant zones of inhibition, ranging from 5 to 25 mm [18,19]. However, the application of synthesised nanoparticles on textiles to develop bioactive fabrics is still challenging.

The present study is focussed on the development of antiviral/antimicrobial, eco-friendly, and hygienic medicated textiles to avoid the spread of hospital-acquired infections. The work involves two major steps. The first step involves the green synthesis of silver nanoparticles, copper nanoparticles, and phytochemicals (having enormous and different medicinal properties) extracted from different parts (leaves, bark, and fruits) of the *Ehretia acuminata* (Family: Boraginaceae) plant. In the second step, the prepared nanoparticles and extracted phytochemicals are loaded on cotton fabrics. The application on fabric is performed using the conventional finishing methodology (pad-dry-cure).

The objectives of the present work are:

➢ The green synthesis of AgNPs by the reduction solution method.
➢ The green synthesis of CuNPs by the reduction solution method.
➢ Extraction of phytochemicals and convert them into micro/nano-size by the ball-milling process.
➢ Study the total yield contents of phytochemicals.
➢ Size reduction enhances the surface area of particles, which in turn enhances the mass transfer of active (antimicrobial agents) agents from plant material to the solvents.
➢ Coating the particles on cotton fabric by the pad-dry-cure method.
➢ Study the effect of different concentrations of silver particles, copper particles, and phytochemicals against pathogens (viruses, bacteria, and fungus).
➢ Study the effect of air permeability and water permeability through particle-coated fabric bandages to ensure the transportation of wound exudates.
➢ Study the durability of coated textiles regarding washing and rubbing actions.

The end use is the development of antibacterial surgical drapes, pants, panels, bed sheets, surgical gowns, curtains, panel covers, wall papers/sheet covers, shoe mats, outlet covers, seat chair covers, table covers, patients’ and doctors’ socks, etc. 

## 2. Materials and Method

### 2.1. Materials

The cotton fabric used in hospital areas (bleached, plane weave, areal density of 150 g per meter square) was obtained from Arif Textile Mills, private limited, Faisalabad, Pakistan.

The fruit, leaves, and bark of *E. acuminata* were obtained from Bagh-e-Jinnah, Lahore, and then submitted to the Botany Department, Government College of Science, Lahore, Pakistan. Tannic acid, sodium hydroxide, binder (LATEX), and silver nitrate were supplied via Sigma Aldrich. DI water was collected from the reagent water system (Milli Q SP, Millipore, Milford, MA, USA) and was utilised during the process of synthesis. All the received chemicals were used without any further purification.

Copper (II) chloride (CuCl_2_·2H_2_O) with 98% purity was obtained from Riedel-de Haen, Germany, and L-ascorbic acid with 99% purity was purchased from Merck, Germany. The chemicals were of analytical grade and used with no further purification or chemical treatment. L-ascorbic acid was used both as a capping and a reducing agent. LB (Luria–Bertani) agar-based broth and Lennox broth were provided by Merck for anti-bacterial testing. All the chemical solutions were prepared freshly for use during all the chemical reactions.

The selection of reducing agent depends upon the ions which are going to be reduced. For the silver nanoparticles, we used tannic acid, which acts as reducing and capping agent. A strong stabilizing agent is required to limit the formation of complex ions of silver during the synthesis process. Secondly, for the reduction of copper ions (due to the transition ionic state of copper +1, +2), a strong reducing agent is required. Lactic acid is a stronger reducing agent compared to tannic acid. Both silver and copper have different surface potentials, so we used different organic materials during their synthesis processes [9]. 

### 2.2. Silver Green Synthesis

**Figure 1:**
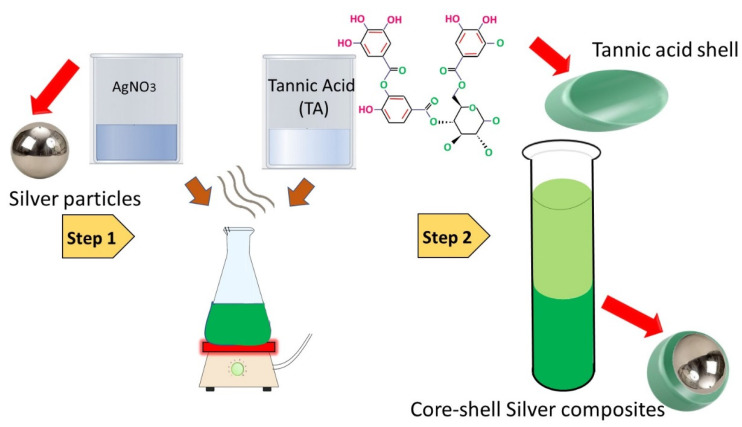
Schematic for the green synthesis of silver nanoparticles.

The green synthesis of silver nanoparticles was executed by one-pot mixing of 0.5 M silver salt AgNO_3_, 1 mM tannic acid (TA), and 0.3 M sodium hydroxide (NaOH) in the ambient environment. The whole assembly was continuously stirred with a magnetic stirrer to homogenize the solution. The speed of stirring was kept constant (150 rpm), and temperature was maintained at 45 °C for 25 min. Subsequently, the solution was cooled down to room temperature and the prepared nanoparticles were centrifuged for purification. The parameters were set at 900 rpm for 20 min and the collected material was washed three times using deionised water. The schematic for the preparation of silver nanoparticles is shown in Figure 1.

### 2.3. Copper Green Synthesis

**Figure 2:**
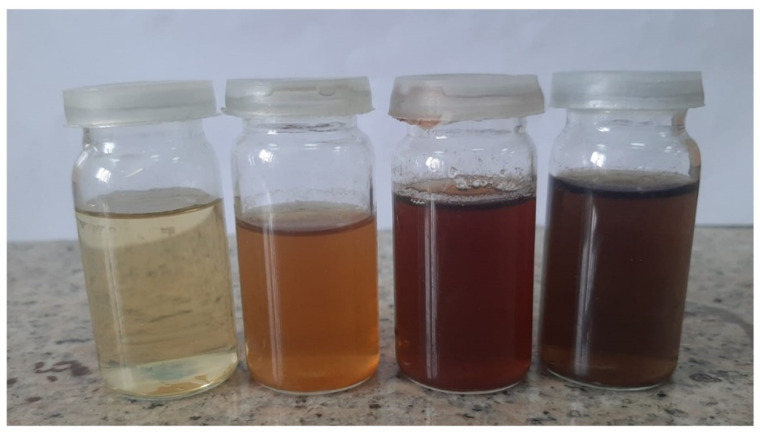
Green synthesised copper nanoparticles and colour change with an increase in the reduction time.

Solution one was prepared via dissolving the copper salt (CuCl_2_·2H_2_O) (0.03 M) in 200 mL of DI water. Solution two was prepared by L-ascorbic acid (1.0 M solution) in DI water, separately. Then, 100 mL of copper chloride solution was added into airtight flasks and heated at 100 °C continuously using a water bath shaker (mechanical/electrical heated), followed by drop-wise addition of 2 molar L-ascorbic acid solution into each flask. The solution was mixed and heated continuously until the colour changed to yellowish, orange, light brown, and finally, chocolate brown colour, as depicted in Figure 2. The completion time of the whole process was 20 h. The final product was then stored for 12 weeks, with no occurrence of dispersion or sedimentation, as checked without any magnification.

### 2.4. Extraction of Phytochemicals

**Figure 3:**
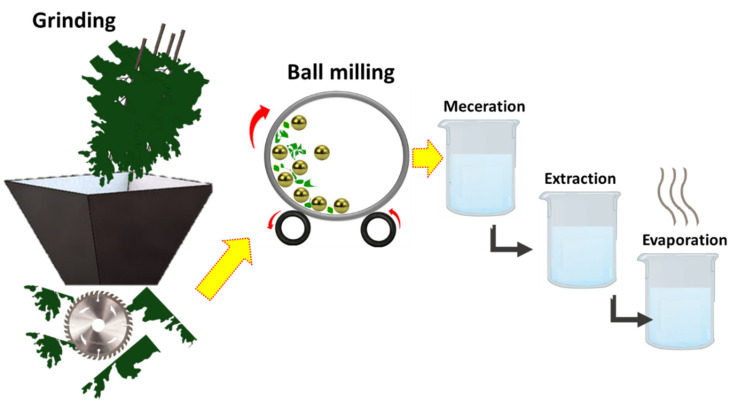
Schematic for extraction and preparation of phytochemicals.

All parts of the plant were washed properly with fresh water to get rid of undesirable substances and kept at room temperature for drying. All dried parts were blended in an electric grinder to convert the long parts into staple fibres. Subsequently, these ground flakes were further refined to micro/nano-scale by using the ball-milling process. High-energy ball-milling (planetary, Fritsch pulverisette 7, Weimar, Germany) was used to carry out dry pulverisation. Zirconium balls (10 mm) and a sintered corundum container (80 mL) were used for drying milling up to 60 min, while the BMR was kept at 10:1 with 700 rpm speed. Then, 400 g of powder of each part, such as leaves, bark, and fruit, was soaked with 1200 mL of Dichloromethane, separately, while 250 g of powder of leaves, bark, and fruit was macerated with 750 mL of methanol for 14 days, with frequent shaking at ordinary temperature. After maceration, it was filtered by using Whatman No.1 filter paper, and the obtained filtrate was subjected to a rotary evaporator to obtain crude extract of the plant material. Resulting crude extracts were stored in vials and placed in a refrigerator at 4 °C. The schematic for extraction and the milling process is shown in Figure 3. 

### 2.5. Pre-Treatment of Cotton Fabric

The cotton fabric was pre-treated before the deposition of the prepared silver, copper, and phytochemical particles on cotton fibres. Pre-treatment was carried out with citric acid. A solution of 20 g/L of citric acid was made and cotton fabric was dipped in it at 90 °C for 2 h, then washed and dried at 90 °C for 50 min.

The purpose of the pre-treatment of cotton fabric with citric acid is to enhance the number of carboxyl groups (–COOH) on the surface of cotton fabric. Moreover, the treatment of cotton with citric acid as a result of the chemicals can produce an ester bond, which initially increases and then tends to stabilize. In a similar study, a shift in the amount of free carboxyl was noticed on the surface of the cotton fabric. The concentration of free carboxyl content in the pristine cotton fabric was around 17 mmol/kg, which is mainly due to the by-products of the oxidative bleaching of the cotton fabric. The amount of free carboxyl groups in the cotton increased (from 16.67 to 727 mmol/kg) with the addition of CA. When the concentration of CA reached a particular point, the fabric surface became saturated, and the amount of free carboxyl groups reached its peak at around 1070 mmol/kg. The variations in the hydroxyl level on the surface of cotton indicate the saturation of the samples [20].

### 2.6. Application of Prepared Particles and Phytochemicals of Cotton Fabric

**Figure 4:**
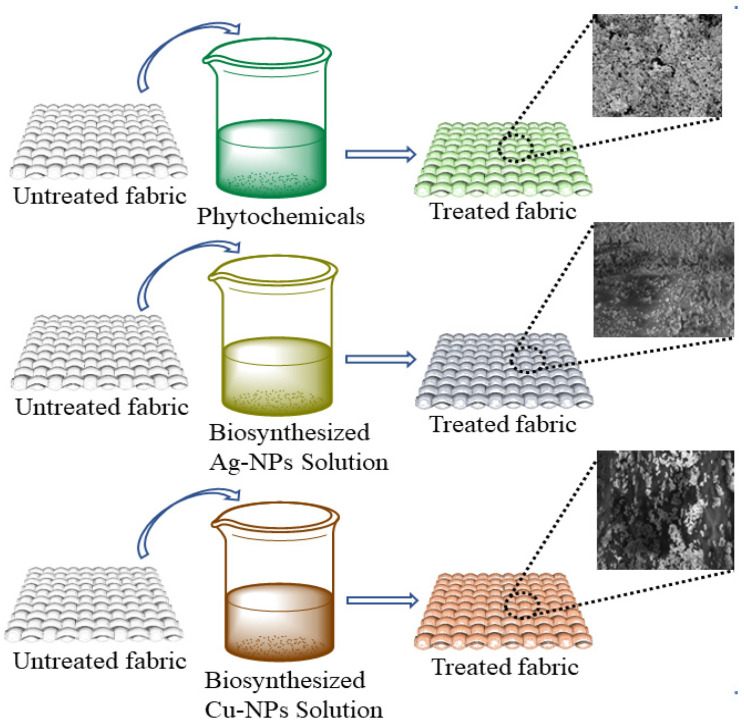
Scheme representing the coating of different types of antimicrobic agents used for the fabrication of the textile.

**Figure 5:**
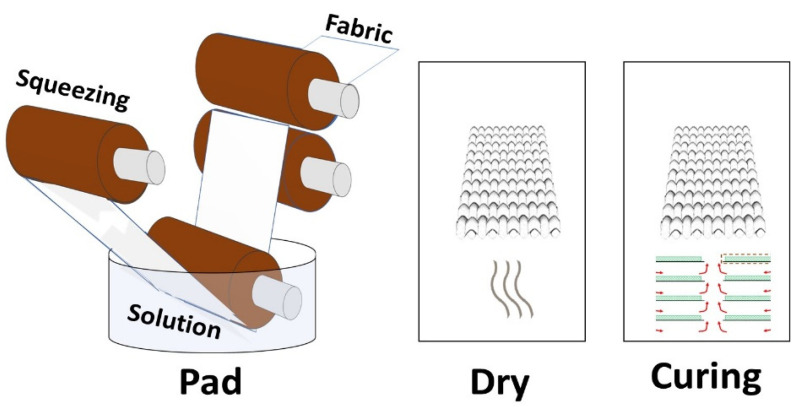
Application of prepared particles and phytochemicals of cotton fabric.

Three different concentrations (0.25 g, 0.5 g, 1 g) of silver particles, three different concentrations (0.25 g, 0.5 g, 1 g) of copper particles, and three different concentrations of phytochemicals (0.25 g, 0.5 g, 1 g) were dissolved in 200 mL of water. Then, 0.5 g of binder was dissolved in each solution. The pH was maintained at 5–6 with the help of citric acid. A 100% wet pick-up was maintained for the treatments. In each solution, the cotton fabric was soaked for 30 min followed by padding and drying at 90 °C for 20 min. The schematic illustration for the coating of different types of antimicrobial agents on cotton fabric is shown in Figure 4. Moreover, the application process of the prepared particles and phytochemicals of the cotton fabric is shown in Figure 5. The design of experiments for the developed samples is presented in Table 1.

### 2.7. Characterisation 

#### 2.7.1. Preliminary Phytochemical Screening

Phytochemical screening of the plant extracts was carried out as per the methods of Kokate, to observe the presence of various phytochemicals [21]:

$$Extractive\;yield=\frac{Mass\;of\;extract}{Mass\;of\;dry\;matter\;}\times 100$$

#### 2.7.2. Characterisation of Surface Morphology

Scanning electron microscopy (SEM, FEI Quanta 50, Hertfordshire, UK) was used for the morphological investigation of silver nanoparticles, copper nanoparticles, and phytochemicals’ deposition on the surface of cotton fabric. XRD analysis was performed using PAN analytical X’pert PRO equipment (Malvern, UK). Dilute dispersion of each particle was prepared using DI water in a beaker followed by its ultrasonication in an ultrasonic bandelin probe before characterisation of particle distribution using the Malvern Zetasizer.

#### 2.7.3. FTIR Analysis of Cotton Fabrics

Infrared spectra both for untreated and treated cotton fabrics were recorded using a Nexus Nicolet 470 spectrometer equipped with an ATR (attenuated total reflection) Pike-Miracle accessory.

#### 2.7.4. Antimicrobial Testing

Qualitative and quantitative measurements were performed for testing the antibacterial activity of cotton fabrics treated with cuprous oxide particles.

#### 2.7.5. Qualitative Test (Zone of Inhibition Measurement)

##### Bacterial Strain Preparation

Gram-positive and Gram-negative bacterial strains viz., *Staphylococcus aureus* (CCM-3953) and *Escherichia coli* (CCM-3954) for this study were obtained from the Microorganism Czech Collection Lab (Masaryk University Brno, Czech Republic). Fresh bacterial suspensions were prepared by growing an overnight single colony within a nutrient bath at 37 °C. Before antibacterial testing, the turbidity of the sample was adjusted to 0.1 optical density at 600 (OD 600). The agar plates were prepared freshly before starting antibacterial tests. The cotton swab (sterilised) was dipped inside the culture suspension and then cells were homogeneously spread on the agar plates. After preparation, these plates were utilised for antibacterial testing [22].

##### Determining Zone of Inhibition

The coated cotton fabrics (6 × 6 mm^2^) with CuO particles were directly placed on inoculated agar plates. The detailed procedures are described in [23]. The untreated cotton fabric used as a control was tested in parallel. The inoculated agar plates and samples were then placed at 37 °C for 24 h. The zone of inhibition was measured as the mm (total diameter) of textile fabric coated with CuO particles along with the zone where there is inhibition of bacterial growth. All experiments were conducted in triplicate and the mean value was calculated.

##### Quantitative Test (Reduction Factor)

Quantitative measurements were performed using the standard AATCC test method (100–2004). It is a quantitative method which uses the reduction factor and explains the percentage reduction of inoculated bacterial concentration due to the sample effect. It results in bacterial colonies survivors (CFU) and then the inhibition degree (%) is calculated from such number. Therefore, it is necessary to perform a comparison of treated and untreated samples (standardised). First, a sample cut in dimensions of 18 × 18 mm^2^ was placed for 30 min in a sterile container. Then, a respective bacterial strain (100 µL) with a 10^5^ CFU/mL concentration was applied for each test. Incubation was performed in a thermoset for 24 h at 37 °C followed by addition of physiological solution (10 mL). After vortexing, a pipette was used for taking 1 mL, and then it was inoculated on a Petri dish using blood agar (for each sample there were inoculated triplets). The result is calculated as the sum of the colonies number of all three dishes [24,25]. 

#### 2.7.6. Antifungal Activity Assessments

The AATCC 100–2004 standard testing protocol was followed to evaluate the antifungal activity of the treated fabric sample. For this purpose, the *Candida albicans* fungal specie was used. Equation (1) was employed to calculate the antifungal activity in terms of the percentage reduction:

(1) $$\mathrm{Percentage}\;\mathrm{reduction}\;R\left(\%\right)=\frac{\left(A-B\right)}{A}*100$$

where *A* and *B* represent the number of spores on control (untreated) and treated cotton samples, respectively.

#### 2.7.7. Antiviral Activity

Behrens and Karber’s method was used for the calculation of virus titres’ reduction from the starting viral titre of infectivity (10^7^). Vero-E6 cultures were grown in Dulbecco’s Modified Eagle Medium (DMEM), which included 9% foetal bovine serum (FBS) and 2% penicillin-streptomycin amphotericin (PSA). The coronavirus infected Vero-E6 cultures at a ratio of 1:3 in polyethylene pots to develop virus strains after one day. The virucidal effect of the produced viral stocks was studied under a microscope. The cell line was combined with 10% FBS and frozen at 90 °C. The supernatant was filtered by moderate centrifugation at 5 to 7 °C at 3700 rpm for 30 min. In the experiment, the supernatant was employed as viral stocks, and all macroscopic residue was removed. To evaluate virus titre, Vero-E6 cell lines were cells that were plated at a density of 2 × 105 in 96-well plates and incubated at standard conditions (24 h at 37 °C) in 6% CO_2_. From 10^1^ through 10^8^, each specimen was diluted 10 times. All dilutions were injected into cell lines and incubated at 6% CO_2_ for 3 days. Behrens and Karber’s protocol was followed for the evaluation of coronavirus titre in cultured cell lines. After that, the control and treated fabric samples of dimensions 20 × 20 mm^2^ were taken and placed in the vials. Viral loads (100 μL) were run through the control and treated fabrics, and viral loads recovered in containers were disinfected using the filter. The coronavirus was diluted from 10^1^ to 10^8^. All dilutions were seeded into Vero-E6 cell lines and incubated at 37 °C in 6% CO_2_ for 3 days. Behrens and Karber’s approach was used to calculate the titres of the coronavirus in the cultured cell lines.

### 2.8. Evaluation of Comfort Properties

Air permeability can be defined as the perpendicular airflow rate to a specific known area. The airflow is maintained under the prescribed differential of air pressure between two material surfaces. The SDL air permeability tester (Per ISO 9237 standard) was used for test performance. The air pressure difference of 100 Pa was set between two substrate surfaces. Moreover, a TH4 bending rigidity tester was used for the stiffness measurement of both untreated and treated cotton fabric.

#### Durability

Durability is measured to check the stability of fabrics in service. For this reason, washing of the coated fabrics was performed according to ISO standard (105-C01). All the samples of fabric were stirred using standard detergent having a 50:1 liquid ratio. Then, samples were stirred for 35 min (600 rpm speed) at 40 °C. Afterward, the fabrics were conditioned and dried in a standard atmosphere for 24 h. The durability was further confirmed with antimicrobial results.

## 3. Results and Discussion

### 3.1. Phytochemicals’ Screening Analysis

The preliminary screening of phytochemicals extracted from each part of the plant (leaves, bark, and fruit) was carried out. The extraction indicated the presence of all active agents, and the results are shown in Table 2. 

The leaf extract contained phytochemicals such as flavonoids, saponins, sugars, phenols, steroids, glycosides, and tannins. In seed extract, only one phytochemical, alkaloids, was absent. Several substances have already been found and reported, including alkaloids, saponins, tannins, sterols, flavonoids, and triterpenoids. Flavonoids have a wide range of antifungal, antiviral, and antibacterial actions. Flavonoids also have antioxidative, cytotoxic, chemo-preventive, and antiproliferative properties. Flavonoids have a fundamental structure derived from the C15 body of flavones, and iso flavonoids have been discovered to be harmful to fungi. Thus, the leaf extract of *M. champaca* exhibited antimicrobial activity due to the presence of flavonoids and other phytochemicals.

### 3.2. Extractive Yield of Leaves, Bark, and Fruit of E. acuminata

The percentage extractions from each part of the plant (leaves, bark, and fruit) against two different solvents is shown below. It is clear that methanolic extraction of phytochemicals was higher as compared to the DCM solvent. It is also noticeable that the amount of phytochemicals was higher in the fruit (3.63% against DCM, and 6.5% methanol). 

Extractive yield of DCM leaves extract = 15.25 gm/400 gm × 100 = 3.8%

Extractive yield of DCM bark extract = 4.60 gm/400 gm × 100 = 1.15%

Extractive yield of DCM fruit extract = 14.50 gm/400 gm × 100 = 3.63%

Extractive yield of methanolic leaves extract = 15.81 gm/250 gm × 100 = 6.32%

Extractive yield of methanolic bark extract = 12.37 gm/250 gm × 100 = 4.95%

Extractive yield of methanolic fruit extract = 16.25 gm/250 gm × 100 = 6.5%

### 3.3. FTIR Analysis

**Figure 6:**
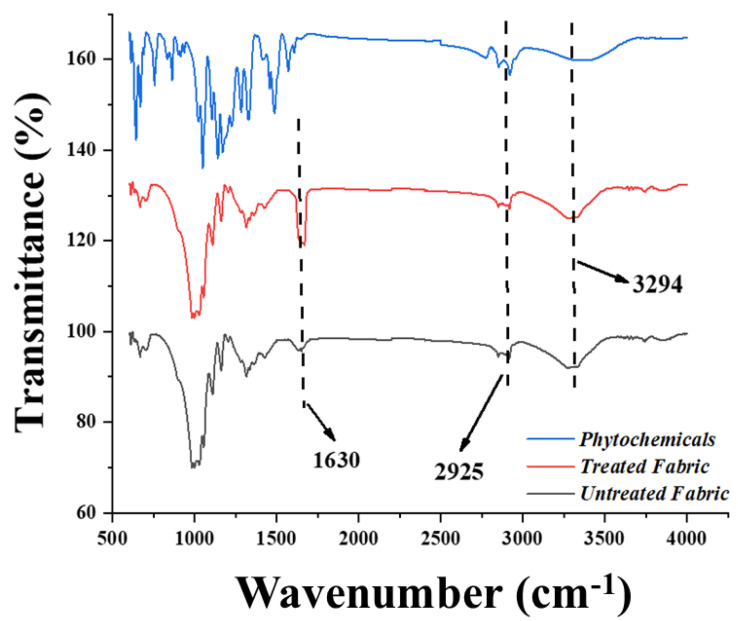
FTIR spectra of untreated cotton, cotton treated with citric acid, and cotton treated with phytochemicals.

Cotton fabric was pre-treated using citric acid to enhance its carboxyl moieties. Based on the assumption, more sites will be provided for silver and copper particles’ attachment with a greater number of added carboxyl groups. Infrared spectra were then recorded using an FTIR spectrometer (Nicolet Nexus 470) equipped with an ATR Pike-Miracle accessory. Figure 6 shows the FTIR spectra obtained for untreated and treated cotton fabric. The broad peak observed at 3300 cm^−1^ corresponds to the stretching of the O-H bond. In contrast, the broad peak observed in the 2800–3000 cm^−1^ region is attributed to C-H stretching. Moreover, for the cotton grafted with citric acid, the absorption band for the carboxylic group appears clearly at 1732 cm^−1^, which is related to the carboxylic acid form [12]. 

Different approaches have been used to investigate and document the phytochemistry of *E. acuminata* (Family: Boraginaceae) [26]. The most important biologically active components include phenolic acids, polyphenolic compounds, and flavonoids (i.e., methyl rosmarinate, free caffeic acid, rosmarinic acid, luteolin-3-glucuronide, luteolin-7-glucuronide, flavones, 6-hydroxyluteolin-7-glucoside, apigenin-7-glucoside, and apigenin-7-glucuronide) [27]. Both alcoholic and aqueous extracts of *Ehretia acuminata* richly contain flavonoids, especially luteolin-7-glucoside and rosmarinic acid [28]. FTIR analysis is a fundamental technique for the detection of functional groups. The appearance of a distinctive peak at 3377 cm^−1^ is attributed to the stretching vibrations of the OH group in polyphenols and the OH group present in sugar rings, whereas the peak observed at 1678 cm^−1^ might be due to the stretching vibrations of the C=O bond inside aromatic rings of various phenolic compounds, which include flavonoids and polyphenols present in the aqueous extract of *Ehretia acuminata*. Meanwhile, the band at 1471 cm^−1^ is correspondent to the stretching mode or vibrations of the C=C group. The occurrence of absorption peaks at 2873 and 2946 cm^−1^ is related to the stretching vibration of C-H methyl groups [29]. Similarly, the peaks observed at 1290 and 1386 cm^−1^ are relative to the strong stretching (C-O) of ester groups and medium bending vibrations of the C-H group, respectively [29]. Furthermore, the absorption bands that appeared at 1253 cm^−1^ are related to the C-O vibration of hydroxy flavonoids [30], while the band at 1162 cm^−1^ corresponds to the strong stretching of the C-O group of tertiary alcohols and the band seen at 1058 cm^−1^ is related to the stretching (C-O) of primary alcohols. In contrast, the absorption bands at 815 cm^−1^ are related to the C-H bending vibration. The major FTIR peaks and associated functional groups are shown in Table 3 [30].

### 3.4. XRD Analysis

**Figure 7:**
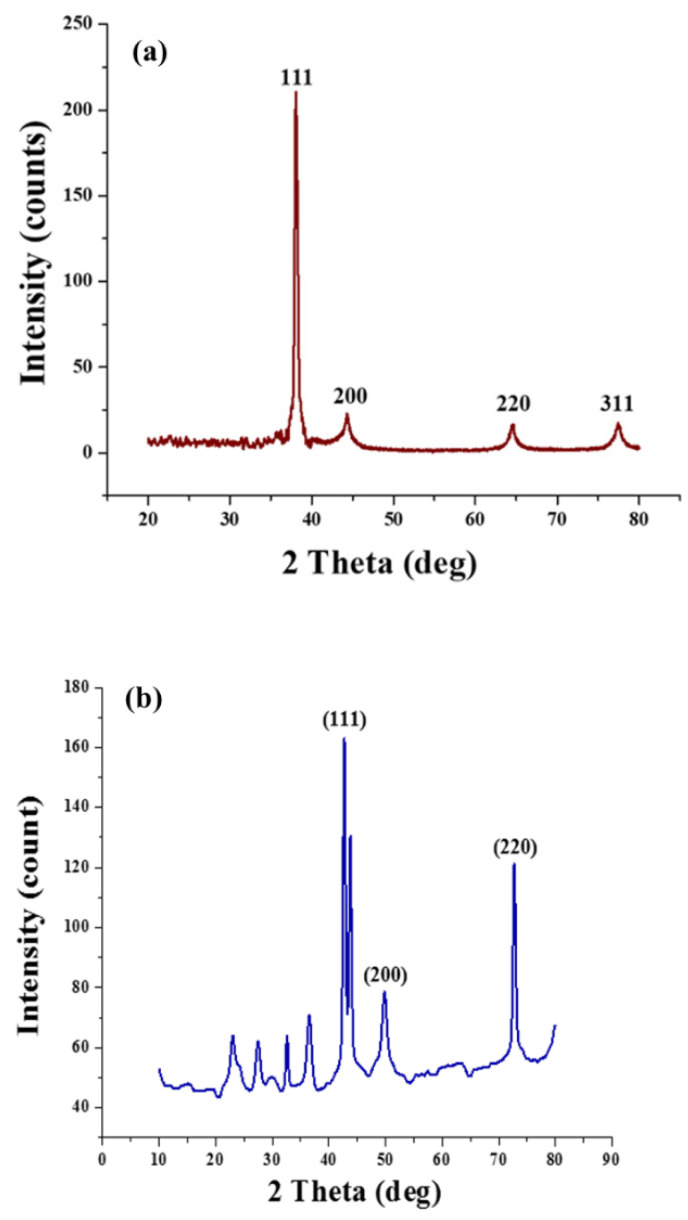
XRD analysis: (**a**) silver-coated cotton and (**b**) copper-coated cotton.

The phase composition of deposited copper and silver nanoparticles was evaluated using XRD analysis (2θ range: 20–80° with a step degree of 0.02°). The phase purity of synthesised Ag nanoparticles can be obvious from the perfect indexing of all the diffraction peaks to the structure of silver, as shown in Figure 7a. As compared to the untreated fabric of cotton, four peaks appeared for silver particles at 2θ values of 77.5, 64.5, 44.3, and 38.1, thus attributed to diffraction planes (3 1 1), (2 2 0), (2 0 0), and (1 1 1), respectively, having a cubic structure as reported in the International Diffraction Centre Data (data number JCPDS 04-0783 card) [13,28]. Moreover, no distinctive peaks were seen for other impurities, such as AgO.

XRD patterns for copper nanoparticles are presented in Figure 7b. The phase purity of copper particles can be clearly seen from the perfect indexing of all the diffraction peaks to the structure of copper. The appearance of copper diffraction peaks (2θ) at 74.2°, 59.5°, and 43.3° represents copper diffraction planes (2 2 0), (2 0 0), and (1 1 1), respectively [29]. The copper particles were examined for their crystalline structure from the appearance of sharp peaks. In contrast, the broadening of the peaks indicated the copper particles’ formation at the nanoscale since no specific impurity peaks were detected, except the appearance of the Cu_2_O peak (2θ) at 38°, respectively [30].

### 3.5. Surface Characterisations

**Figure 8:**
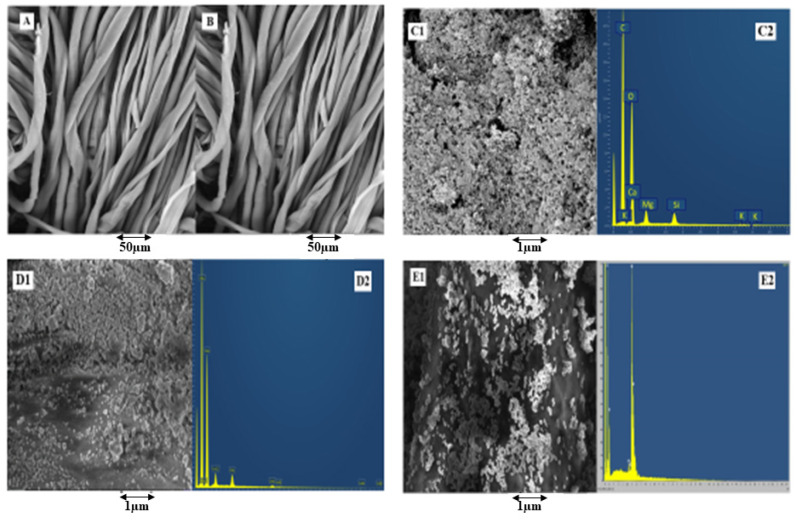
SEM analysis of (**A**) untreated cotton, (**B**) cotton treated with citric acid, (**C1**) phytochemicals-coated, (**D1**) copper particle-coated, and (**E1**) silver particle-coated cotton fibres, with EDX spectra of (**C2**) phytochemical-coated, (**D2**) copper particle-coated, and (**E2**) silver particle-coated cotton fibres.

SEM analysis was performed at each step. Figure 8A shows the SEM analysis of simple cotton fibres without any treatment and coating. Figure 8B shows the microstructures after treating with citric acid. The treatment was performed to enhance the carbonyl groups on the surface of cotton cellulosic structures. The treated fibre surface condition showed minor roughness as compared to simple untreated cotton fibres.

To check the successful deposition of phytochemicals, copper (Cu), and silver (Ag) nanoparticles on the surface of a fabric, SEM analysis was performed. SEM images in Figure 8D1,E1 show the deposition of Cu-NPs and Ag-NPs in the nanometric range. The nanoparticles’ deposition was found to be denser and uniform, whereas the size of the phytochemical particles extracted showed that the prepared particles lie in the micro- to the nano-range. The morphological aspects of particles showed them as rough surfaces with irregular clusters and quasi-spherical shapes along with small agglomeration. Moreover, microparticles can be seen as evenly distributed on the surface of a fabric. It is emphasised that agglomeration was not seen on the larger portion of prepared materials, as evident from Figure 8C1. 

Table 4 shows the elemental composition for fabrics coated with phytochemicals, silver, and copper, respectively. From the table, it can be observed that copper and silver contents increased with the increase in concentration. To gather more facts regarding composition and the elemental presence within phytochemicals, EDX spectral analysis was conducted to find out the elemental composition. The oxygen (O) and carbon © peaks are attributed to the presence of phytochemicals on the cotton fibre surface. In all such elements, the presence of oxygen and carbon is in a high concentration, while calcium, magnesium, and silicon are found to be in the moderate range. Trace elements are determined in trace quantities through the percentage (%) abundance of elements such as Ca, K, Cl, Si, and Mg within the sample.

### 3.6. Particle Size Distribution

**Figure 9:**
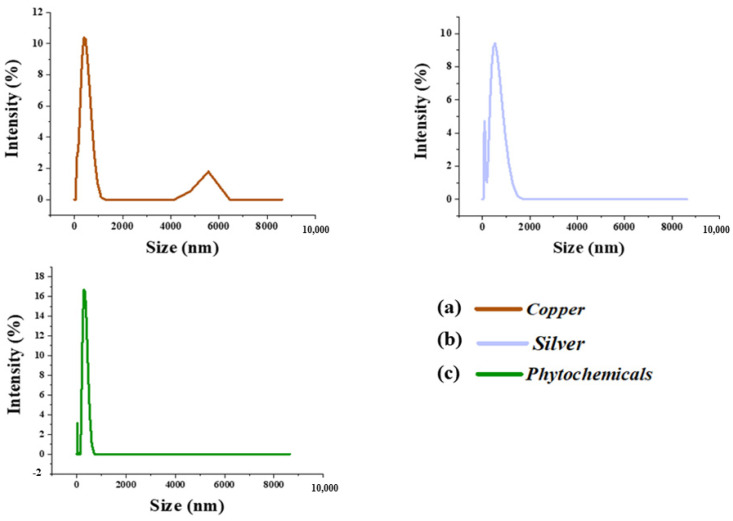
The particle size distribution of (**a**) copper, (**b**) silver, and (**c**) phytochemical particles.

Based on the Brownian movement of particles, the DLS technique was used for particle size measurement. Figure 9a,b reveal the particle size distribution of copper and silver particles. The particles were known to exhibit multi-modal distribution with size-changing from mm to nm. The average particle sizes found for silver and copper were 600 and 500 nm, respectively. The particle size of the extracted phytochemicals was found to be from the nano- to the micro-range. The average particle size and zeta potential of the extracted phytochemicals were about 312 nm and −41.6 mV, as shown in Figure 9. The particles were found to be in the nano–micro regime with the highest negative potential, which proved the even distribution and stability of the nanoparticles in the suspension.

### 3.7. Antibacterial Activity of the Coated Fabrics

The antibacterial activity of all coated fabrics was tested against qualitative and quantitative measurements. 

#### 3.7.1. Zone of Inhibition Test (Qualitative Measurements)

**Figure 10:**
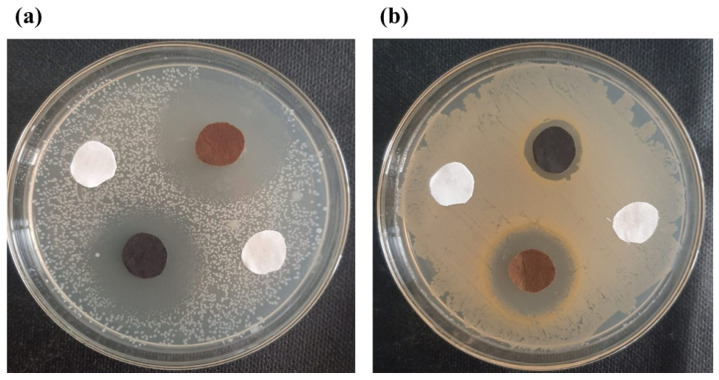
Inhibition zones against (**a**) *S. aureus* and (**b**) *E. coli*.

**Figure 11:**
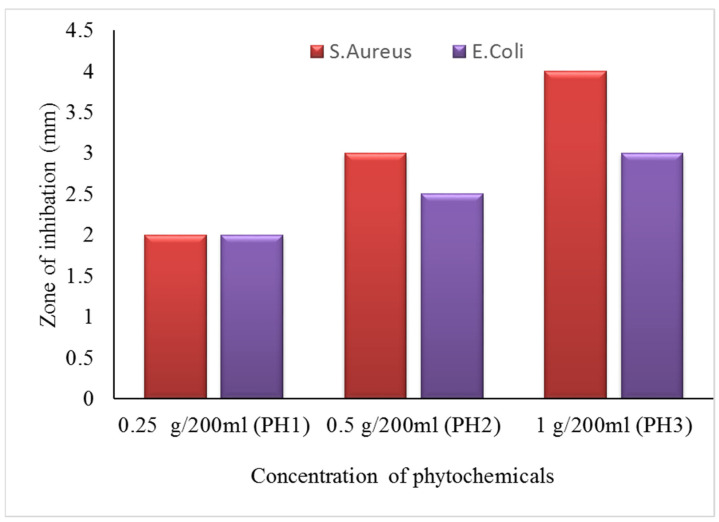
The average value of the zone of inhibition against phytochemicals.

**Figure 12:**
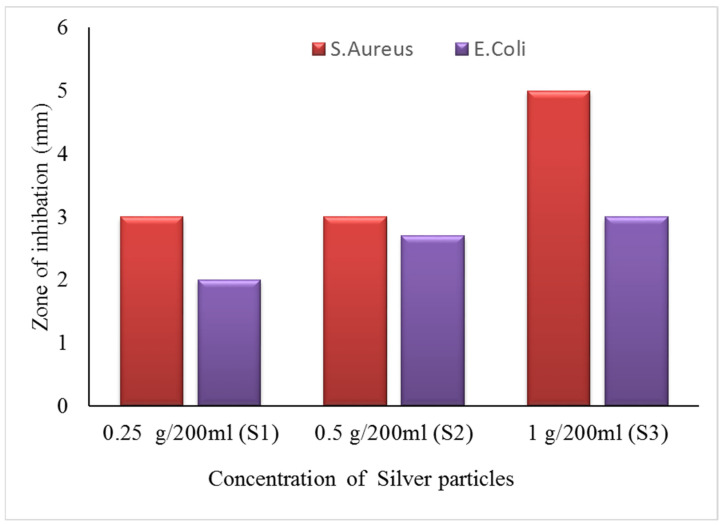
The average value of the zone of inhibition against silver particle-coated cotton fabric.

**Figure 13:**
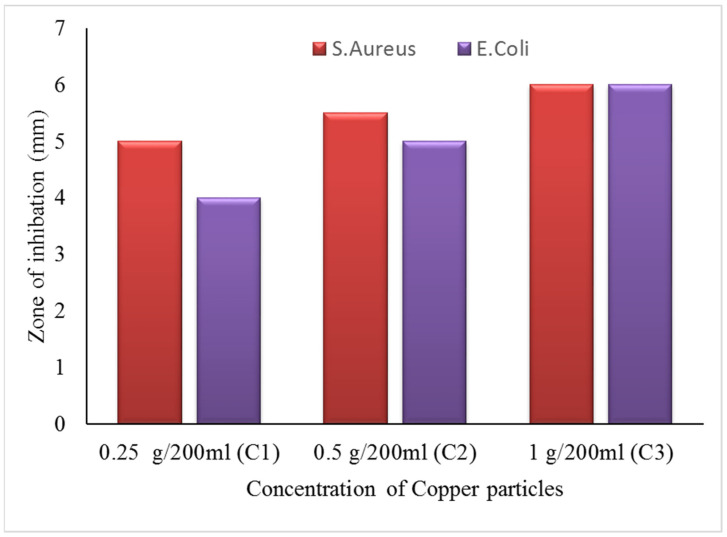
The average value of the zone of inhibition against copper particle-coated cotton fabric.

The zone of inhibition test is considered a qualitative assessment. The test was conducted against both bacterial types, Gram-positive (*S. aureus*) and Gram-negative (*E. coli*). Clear inhibition zones around all types of fabric samples incubated for 24 h at 37 °C in the dark are represented in Figure 10. It is obvious from the figures that fabrics coated with phytochemical particles show less zone of inhibition, whereas the copper (green synthesis)-coated fabrics revealed the most significant antibacterial zones against *S. aureus* and *E. coli* bacterial strains. Three repetitive tests were conducted against each sample and then the average value was calculated for each agent, as shown in Figure 11, Figure 12 and Figure 13. The results confirmed that deposited copper and silver particles induce strong sterilisation towards *S. aureus* and *E. coli* owing to the free-standing nature of particles. However, the highest sensitivity is depicted by *S. aureus* in comparison to *E. coli.* The increase in the zone of inhibition for *S. aureus* was from 4 to 5 mm, whereas it increased from 3 to 4.5 mm for *E. coli* with an increase in the concentration of copper particles. It is worth mentioning here that the annulus of the zone of inhibition increases with an increase in the load particles’ concentration, which indicates that a higher content of nanoparticles will affect the antibacterial activity, similar to the previous study [8]. 

The antibacterial activity of coated fabrics can be credited to the combined physical and chemical interaction of bacteria with particles. Nanoparticles can be added to the cell through endocytotic mechanisms. Then, the ion’s cellular uptake increased as ionic species were subsequently released in the cells through nanoparticle dissolution [31]. This resulted in a higher intracellular concentration inside cells for further enormous oxidative stress. 

#### 3.7.2. Reduction Factor (Quantitative Test)

**Figure 14:**
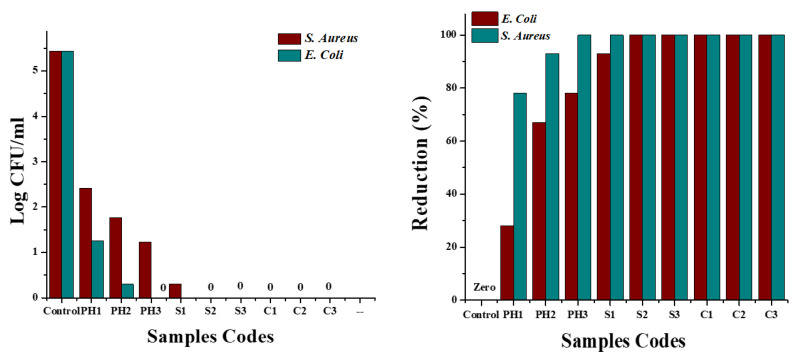
Antibacterial activity in terms of log CFU/mL (**left**) and percentage reduction (**right**) of fabrics treated with phytochemicals, silver, and copper nanoparticles and untreated cotton fabric.

For quantitative measures, the AATCC-100 method was adopted, and samples were tested against both *S. aureus* and *E. coli* bacterial strains. The antibacterial action of all samples regarding reduction in terms of log CFU/mL and percentage reduction calculated from log CFU/mL values are shown in Figure 14. The figure on the left side depicts the reduction in inoculated bacterial concentration (log CFU/mL). It was observed that log values were significantly reduced for all treated samples as compared to log values of untreated fabric, and the reduction in the log value was increased with the increase in concentrations of nanoparticles (copper and silver) on fabric. In the case of samples loaded with the highest concentrations of copper and silver nanoparticles, the log value decreased from 5.43 to 0, suggesting a 99.99% reduction in inoculated bacterial colonies (figure on the right side, Figure 14) for both *S. aureus* and *E. coli*. The log CFU/mL value for untreated cotton fabric was increased from 5.43 to 5.44, revealing ineffectiveness of the control sample against both tested microbes. The reduction percentage of untreated and treated cotton fibres is shown in Table 5.

**Figure 15:**
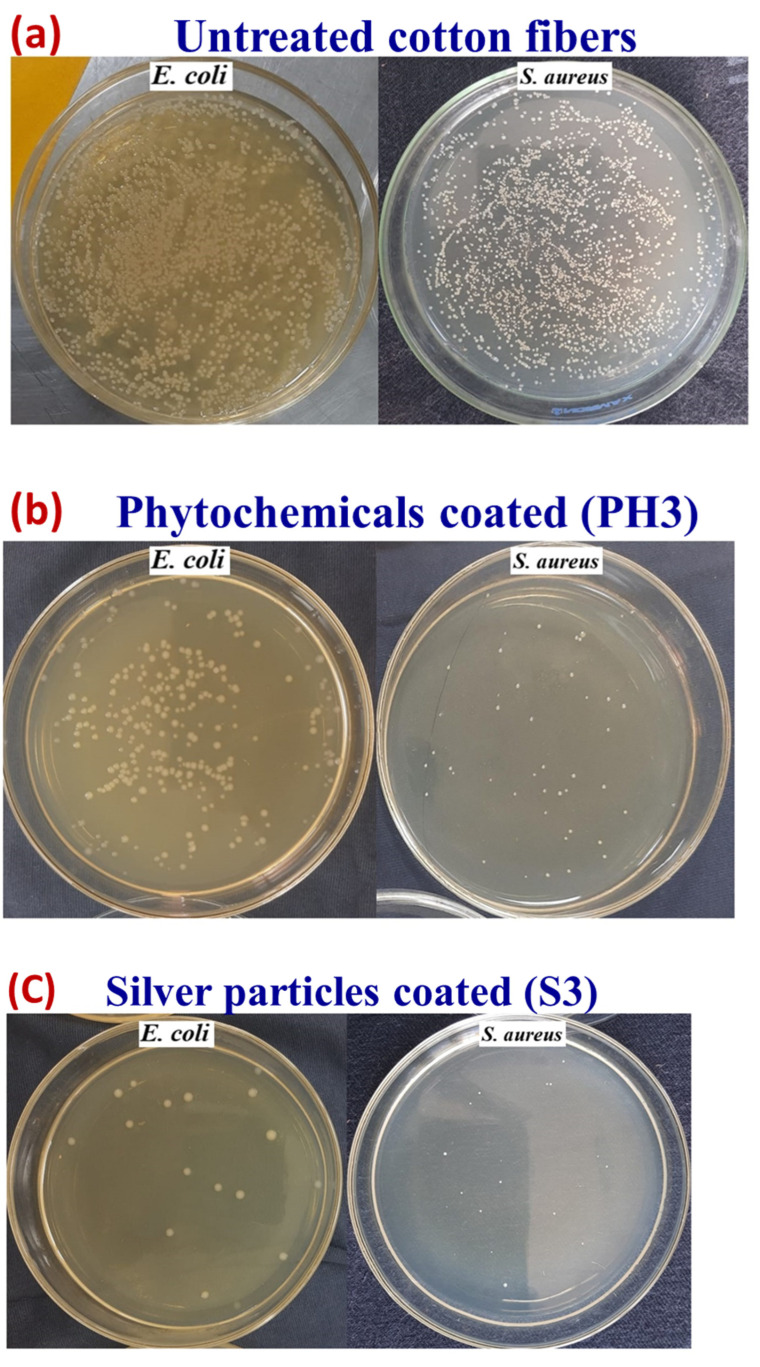

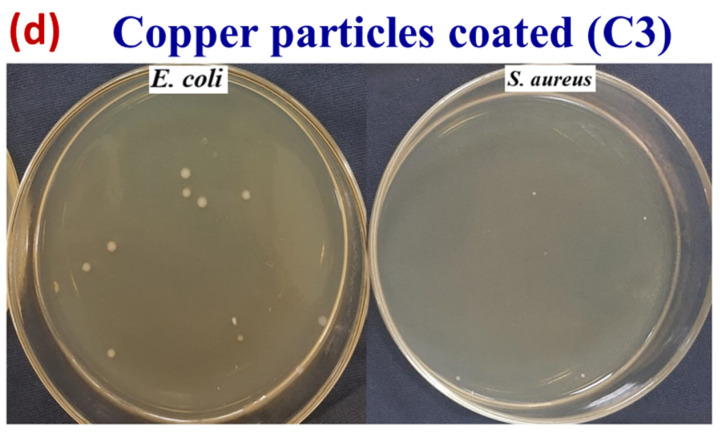
Images of concentration of bacterial growth for the (**a**) untreated (pristine) cotton fabric, (**b**) for phytochemicals, (**c**) for silver particles, and (**d**) for copper particles.

Figure 15 shows selected pictures of bacterial growth concentration for untreated cotton fabric and treated (PH3, C3, and S3) samples, which support the above trend. The untreated sample was shown to be ineffective against bacterial growth, whereas the textiles coated with copper particles were found to be highly effective. However, with a higher concentration of copper particles, there was a significant improvement in colony reduction with an effectiveness of greater than 99% for both types of bacteria.

### 3.8. Antifungal Activity of Treated Samples

The antifungal activity was evaluated according to the standard test method of qualitative measurement. The fungus *C. albicans* was selected for this purpose. The activity was checked for all phytochemical-, silver particle-, and copper particle-coated cotton fibres. The percentage reduction of fungi (*C. albicans*) with raw cotton and samples loaded with copper, silver, and phytochemicals is presented in Table 6. The raw cotton fabrics without any antifungal agent can provide a suitable environment for microorganisms to grow. The copper particle-coated cotton fibres showed maximum antifungal activity (as copper particle-coated fibre also showed the best antibacterial properties). The percentage reduction of silver nanoparticles was also higher as compared to phytochemicals. 

### 3.9. Antiviral Effectiveness

**Figure 16:**
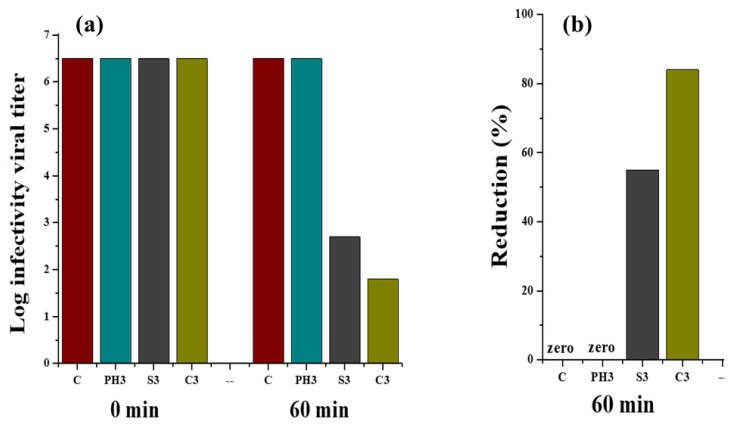
Reduction in viral infectivity titre (**a**) and percentage reduction (**b**) calculated from viral infectivity for untreated and treated fabrics at a contact time of 0 and 60 min.

The graph in Figure 16 shows the virus infectivity titre log against contact time (0 h and 60 min). Behrens and Karber’s method was used for the calculation of virus titres’ reduction from the starting viral titre of infectivity (10^7^). Figure 16a shows the infectivity titre change of coronavirus (0 h and 60 min) at 25 °C for untreated cotton fabric and fabrics treated with phytochemicals, copper, and silver nanoparticles. It was observed that there was a significant decrease in the infectivity titre for fabrics coated with copper and silver particles after 60 min as compared to 0 h, whereas no reduction in the virus activity titre was observed in case of phytochemical-treated fabric and untreated cotton fabric. Similarly, Figure 16b depicts the virus percent reduction for untreated and treated fabrics. The fabrics treated with copper and silver showed 84% and 55% reductions in virus titre, respectively, whereas phytochemical-treated fabric and untreated fabric remained ineffective against the virus. The antiviral action shown by the fabrics treated with copper and silver nanoparticles could be due to the binding of metallic NPs with glycoproteins at the viral surface, working as an inhibitory action for viruses.

### 3.10. Air Permeability

Air permeability is the exchange of air when heat and perspiration are generated from a wound [32]. The results of fabric air permeability are shown in Table 7. The air permeability results were found for all types of fabrics (treated and untreated). 

From the results, it is clear that the application of very fine nanoparticles to the fabrics had very little effect on air permeability. Air permeability of the untreated fabric was about 142 mm/s, while the air permeability for all other nanoparticle-coated fabrics was in the range of 129 to 136 mm/s, showing that there was a minor decrease in air permeability even after depositing the nanoparticles. There are two factors responsible for this phenomenon. Firstly, there may be a relaxation shrinkage in the fabric structure due to dipping in the nanoparticle solution, causing the yarns to come close and hinder the flow of air. Secondly, nanoparticles have deposited on the yarn structure and interstices and reduce the fabric air gap spacing (pore size). 

The stiffness of fabric describes its ability to resist deformation and keep standing without support. This property is important regarding comfort and desirable draping. Stiffness can be calculated from the bending length and flexural rigidity. The stiffness of coated and uncoated fabric samples was found, and average values are presented in Table 7. Fabric stiffness was found to increase with the increase in the concentration of particles. The reason is that the coating increased the inter-fibre friction and abrasion at fibre crossover points [33]. However, the effect of the increase in rigidity overall was insignificant. 

### 3.11. Durability against Washing

**Figure 17:**
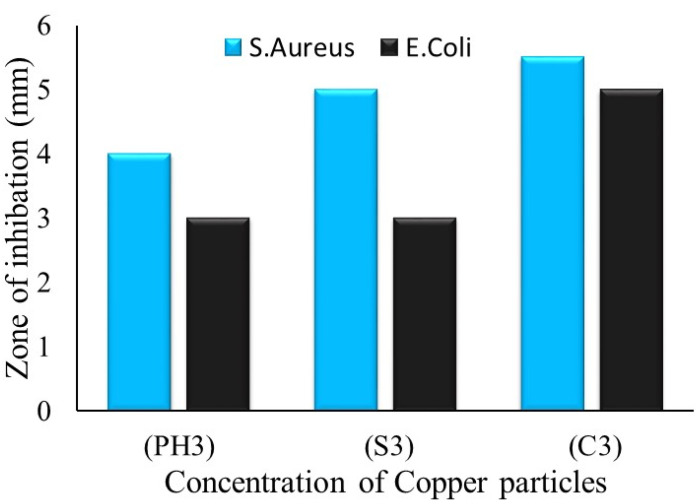
Antibacterial activity of coated fabrics after washing.

The durability of conductive fabrics under washing and rubbing has been a critical challenge. To investigate these properties, samples PH3, S3, and A3 were selected. These three samples were selected because they provided satisfactory results regarding antibacterial properties. A standard washing of these three samples was performed. The antibacterial activity was again checked for the washed samples and reported in Figure 17. There was an insignificant decrease in antibacterial properties. The reason is that the coating of antibacterial agents is so condensed and compact. The retention of the coating and particles was present on the fibrous surface even after severe washing (20 washing cycles). 

## 4. Conclusions

The copper- and silver-coated anti-pathogenic textiles have already been extensively studied due to their potential applications in hospitals. However, to overcome the hygienic issues (mostly occurring due to synthetic antibacterial agents), the present study was focused on a sustainable approach for the green synthesis of silver nanoparticles, green synthesis of copper nanoparticles, and the investigation of the phytochemical and biological screening of bark, leaves, and fruits of *Ehretia acuminata* (belongs to the family Boraginaceae). Surface morphology, size, and the presence of antimicrobial agents (phytochemicals and particles) were analysed by scanning electron microscopy, dynamic light scattering, and energy-dispersive X-ray spectroscopy. The functional groups and presence of particles (copper and silver) were found by FTIR and XRD analysis. The antibacterial activity of coated fabrics was tested against qualitative and quantitative measurements. All the particles showed excellent reduction percentages, but in case of qualitative measurements, the phytochemicals showed no activity. The overall strongest antibacterial effect was found for the fabrics coated with green synthesised copper particles (zone of inhibition about 5 mm and quantitative reduction percentage 99.99%). The coated cotton fibres were further investigated for antiviral and antifungal analysis. There was a significant decrease in the infectivity titre for fabrics coated with copper and silver particles after 60 min as compared to 0 h, whereas no reduction in virus activity titre was observed in case of phytochemical-treated fabric and untreated cotton fabric. The fabrics treated with copper and silver showed 84% and 55% reductions in virus titre, respectively. Furthermore, the copper particle-coated cotton fibres showed maximum antifungal activity (as copper particle-coated fibre also showed the best antibacterial properties). The percentage reduction of silver nanoparticles was also higher as compared to phytochemicals.

Moreover, the developed fabrics were analysed for comfort properties regarding air permeability and stiffness. The particles were so fine that they did not block the pores of fabrics, and hence we ensured the air permeability through the structure, while stiffness was also improved. The discharge of toxic metals from the treated textiles through wastewater has a negative impact on the environment due to their biocidal effect. If the concentration of metals in wastewater is higher than their MIC, they will kill the microbes necessary for the sewage biodegradation. In case of a concentration lower than their MIC, these metals will induce resistance towards those microbes, which is also highly undesirable. The fabrics developed in this study were highly durable to washing (20 industrial washing cycles) and did not release toxic metals into the environment during washing. Therefore, the present study was also focused on the development of durable antibacterial fabrics where the particles (copper, silver, phytochemicals) are firmly attached to the fabric surface (less leaching into the environment), having a zone of inhibition in the range of 3–5.5 mm. The antibacterial activity of treated fabrics after washing was studied to establish the washing durability of the deposition. The finding that particles are strongly adhered to the fibres and interspaces was further supported by the fact that particles remained attached to the surface of fabric during washing. Furthermore, by comparing copper and silver, the antibacterial effect of copper was higher than silver due to the transition in its ionic states. The proposed approach is facile, cost-effective, and offers odourless workwear. The developed antibacterial fabrics could be effectively applied in the field of hospital textiles for the fabrication of antibacterial surgical gowns, panel covers, bed sheets, coveralls, curtains, table and chair covers, etc.

## Figures and Tables

**Table 1:** Design of experiments for the developed samples.

No. of Samples	Agent	Code of Sample	Concentration of Particles
1	Simple cotton fibres	Untreated	0
2	Phytochemicals	PH1	0.25 g/200 mL
3	Phytochemicals	PH2	0.5 g/200 mL
4	Phytochemicals	PH3	1 g/200 mL
5	Silver nanoparticles	S1	0.25 g/200 mL
6	Silver nanoparticles	S2	0.5 g/200 mL
7	Silver nanoparticles	S3	1 g/200 mL
8	Copper nanoparticles	C1	0.25 g/200 mL
9	Copper nanoparticles	C2	0.5 g/200 mL
10	Copper nanoparticles	C3	1 g/200 mL

**Table 2:** Phytochemicals present in seeds, leaves, and bark.

	Phytochemicals	Seeds	Leaves	Bark
1	Flavonoids	+	+	+
2	Phenols	+	+	+
3	Steroids	+	+	+
4	Terpenoids	+	+	+
5	Tannins	+	+	+
6	Glycosides	+	+	+
7	Alkaloids	−	+	+
8	Carbohydrates	+	+	+
9	Protein	+	+	+

Note: Present +, Absent −.

**Table 3:** Major FTIR peaks and associated functional groups [30].

Peaks	Functional Groups
3294 cm^−1^	Hydroxyl group (-OH) stretching vibrations
2925 cm^−1^	Aromatic -CH
1630 cm^−1^	Carbonyl (C=O) stretching vibrations
1200–1300 cm^−1^	Aromatic -CH bending vibrations
1000–1100 cm^−1^	Phenolic (C-O-H stretching vibrations)

**Table 4:** Elemental composition for fabrics coated with phytochemicals, silver, and copper, respectively.

Atomic %	C	O	Si	Ca	Cu	K	Mg	Ag	Total
Phytochemicals on cotton	60.11	35.96	0.68	0.45	-	2.35	0.45		100.00
Copper on cotton	59.86	33.54	1.18	0.33	5.08				100.00
Silver on cotton	51.74	36.09	-	-	-	1.50	-	10.66	100.00

**Table 5:** Reduction percentage of untreated and treated cotton fibres.

Sample	*Escherichia coli*	*Staphylococcus aureus*
	Result, % Inhibiton	Result, % Inhibiton
Untreated standard	0%	0%
Phytochemicals (PH1)	28.69%	78.99%
Phytochemicals (PH2)	67.9%	93.99%
Phytochemicals (PH3)	78.2%	99.99%
Silver nanoparticles (S1)	93.49%	99.99%
Silver nanoparticles (S2)	99.99%	99.99%
Silver nanoparticles (S3)	99.9%	99.99%
Copper nanoparticles (C1)	99.99%	99.97%
Copper nanoparticles (C2)	99.99%	99.99%
Copper nanoparticles (C3)	99.99%	99.99%

**Table 6:** Reduction percentage of antifungal activity.

No. of Samples	Agent	Code of Sample	Concentration of Particles	Reduction %
1	Simple cotton fibres	Untreated	0	0
2	Phytochemicals	PH1	0.25 g/200 mL	0
3	Phytochemicals	PH2	0.5 g/200 mL	6.34
4	Phytochemicals	PH3	1 g/200 mL	22.15
5	Silver nanoparticles	S1	0.25 g/200 mL	23.34
6	Silver nanoparticles	S2	0.5 g/200 mL	42.63
7	Silver nanoparticles	S3	1 g/200 mL	61.45
8	Copper nanoparticles	C1	0.25 g/200 mL	45.29
9	Copper nanoparticles	C2	0.5 g/200 mL	75.45
10	Copper nanoparticles	C3	1 g/200 mL	78.56

**Table 7:** Comfort properties of untreated and coated fabric samples.

No. of Samples	Agent	Code of Sample	Concentration of Particles	Air Permeability (m/s)	Stiffness (N.m)
1	Simple cotton fibres	Untreated	0	142	111
2	Phytochemicals	PH1	0.25/200 mL	136	114
3	Phytochemicals	PH2	0.5 g/200 mL	135	113
4	Phytochemicals	PH3	1 g/200 mL	131	109
5	Silver nanoparticles	S1	0.25/200 mL	134	115
6	Silver nanoparticles	S2	0.5 g/200 mL	135	113
7	Silver nanoparticles	S3	1 g/200 mL	129	110
8	Copper nanoparticles	C1	0.25/200 mL	135	114
9	Copper nanoparticles	C2	0.5 g/200 mL	134	114
10	Copper nanoparticles	C3	1 g/200 mL	133	104

## Data Availability

Not applicable.
